# Body Mass Index in Young Adulthood and Suicidal Behavior up to Age 59 in a Cohort of Swedish Men

**DOI:** 10.1371/journal.pone.0101213

**Published:** 2014-07-01

**Authors:** Alma Sörberg, David Gunnell, Daniel Falkstedt, Peter Allebeck, Maria Åberg, Tomas Hemmingsson

**Affiliations:** 1 Institute of Environmental Medicine, Karolinska Institutet, Stockholm, Sweden; 2 School of Social and Community Medicine, University of Bristol, Bristol, United Kingdom; 3 Department of Public Health Sciences, Karolinska Institutet, Stockholm, Sweden; 4 Division of Psychology, Department of Clinical Neuroscience, Karolinska Institutet, Stockholm, Sweden; 5 Center for Brain Repair and Rehabilitation, Institute of Neuroscience and Physiology, University of Gothenburg, Gothenburg, Sweden; 6 Department of Primary Health Care, Institute of Medicine, University of Gothenburg, Gothenburg, Sweden; 7 Centre for Social Research on Alcohol and Drugs, Stockholm University, Stockholm, Sweden; University Hospital of Bellvitge-IDIBELL; CIBER Fisiopatología Obesidad y Nutrición (CIBERObn), Instituto Salud Carlos III; Department of Clinical Sciences, School of Medicine, University of Barcelona, Spain, Spain

## Abstract

An association of higher body mass index (BMI) with lower risk of attempted and completed suicide has been reported. In contrast, increasing BMI has been found to be associated with depression and other risk factors for suicidal behavior. We aimed to investigate this possible paradox in a cohort comprising 49 000 Swedish men. BMI, mental health, lifestyle and socioeconomic measures were recorded at conscription in 1969–70, at ages 18–20. Information on attempted suicide 1973–2008 and completed suicide 1971–2008 was obtained from national records. Hazard ratios (HR) were estimated by Cox proportional hazard models. We found that each standard deviation (SD) increase in BMI was associated with a 12% lower risk of later suicide attempt (HR 0.88, 95% CI 0.83–0.94). Associations were somewhat weaker for completed suicide and did not reach conventional levels of statistical significance (HR 0.93, 95% CI 0.85–1.01). Adjustment for a wide range of possible confounding factors had little effect on the associations. Lower BMI at conscription was also associated with higher prevalence of psychiatric diagnoses, low emotional control and depressed mood. Our results confirm previous findings regarding the association of higher BMI with a reduced risk of suicide, extending them to show similar findings in relation to suicide attempts. The associations were little affected by adjustment for a range of possible confounding factors. However, we found no evidence that high BMI was associated with an increased risk of depression cross-sectionally or longitudinally.

## Introduction

Several prospective studies have found an inverse association between body mass index (BMI) and suicide, even after adjustment for confounders such as socioeconomic position [1], [2], psychiatric and somatic conditions [1], [2], [3], [4], [5], physical activity [4], [5], [6] and marital status [3], [5], although contradictory findings exist [7]. Suicide attempt is less investigated in prospective studies [8], [9] but the largest study to date found a similar inverse association [9]. This might seem paradoxical, since higher BMI is associated with depression and other mental health risk factors for suicidal behavior in some studies [10], [11]. In the Norwegian HUNT-study, following 74 000 men and women for 17 years, higher BMI was associated with higher risk of depression, but lower risk of suicide, suggesting different mechanisms for the two outcomes despite their interrelation [6]. However, others have found inverse associations between BMI and indicators of mental ill-health [12], [13], [14], or a U-shaped association with elevated risks among underweight as well as overweight persons [15], [16]. Moreover, lower weight has been associated with schizophrenia [17], [18], which is also a risk factor for suicidal behavior.

Some potential explanations for the lower risk of suicidal behavior with higher BMI have been suggested [19], [20]. For instance, lower BMI might predispose to the mental illnesses associated with suicide risk. Confounding by pre-existing mental illness or other risk factors for suicidal behaviors, such as low socioeconomic position and substance use, have also been suggested but has limited support in the literature [19], [20]. BMI has also been found to be associated with certain personality aspects including neuroticism [21], which in turn is associated with an increased risk of suicidal behavior [22]. Furthermore, since BMI has been shown to be associated with adult socioeconomic position and marital status [23], [24], [25]; those factors might mediate the relationship. The few previous studies with BMI data objectively measured in youth have not controlled for substance use, later psychiatric diagnoses, or marital status in adulthood.

This study addresses the following questions: 1. Is BMI in young adulthood associated with later attempted and completed suicide? 2. If so, to what extent is the association explained by socioeconomic background, emotional control, depressed mood, psychiatric illness and substance use in adolescence, and psychiatric illness, socioeconomic position and marital status in adulthood?

We used a cohort of 49 321 Swedish men, born in 1949–1951, who were conscripted for military service in 1969–1970. Data collected on individual cohort members included objective measures of height and weight, full medical examinations, assessments of emotional control, and self-reported substance use and depressed mood. Information on socio-economic conditions, and psychiatric diagnosis and marital status in adulthood was obtained from national registers. Women were not obliged to participate at conscription, and consequently the cohort includes men only.

## Method

### Ethics statement

Ethical approval was obtained from Stockholm's Regional Ethical Review Board at Karolinska Institutet, decision reference numbers 2004/5:9–639/5 and 2010/604:32. Due to the character of the database and the anonymization of all data, the Review Board waived the normal requirement for written consent.

### Study population

The study was based on a cohort of 49 321 young Swedish men aged 18–20, who were conscripted for compulsory military service in 1969 and 1970. The background of the Swedish conscription surveys and the variables included have been presented in detail elsewhere [26], [27], [28]. Only 2–3% of all Swedish men were exempted from conscription; in most cases due to severe handicaps or congenital disorders. Ninety-eight per cent of all men conscripted in 1969 and 1970 were born in 1949–1951; the remaining 2% were born before 1949 and were excluded from this analysis to maximize the homogeneity of cohort members.

### Exposure variable: BMI

Measures of height and weight at conscription were used to calculate BMI (weight divided by height squared) and categorized according to the WHO classifications of underweight (<18.5), normal weight (18.5–25), overweight (25–30) and obesity (>30). Percentiles of BMI (5th, 10th, 25th, 75th, 90th and 95th percentiles, using 25–75th percentiles as the reference group) were also calculated (tables S1 and S2). Information on height and weight was available for 48 904 (99.2%) of the men.

### Outcome variables: attempted and completed suicide

Information on completed suicide 1971–2008 was obtained from the National Cause of Death Register, and information on attempted suicide from the Swedish Hospital Discharge Register, for which data was available for 1973–2008 and therefore has a slightly shorter time span for follow-up. Suicide as well as suicide attempt was classified through the following diagnoses: intentional suicide and harm, E950-9 (ICD-8/9) and X60–X84 (ICD-10); undetermined events, E980-9 (ICD-8/9) and Y10–Y34 (ICD-10). Deaths of undetermined intent were included as has been done previously [1], [6], [9], [29], [30]; most of the undetermined events are likely to be suicides [31], [32]. The same codes were used to identify hospital admissions for suicide attempts, including men kept at least overnight at the hospital.

### Covariates (potential explanatory factors)

#### Factors in childhood, age 9–11

Information on socioeconomic position and crowded housing in the conscripts' childhood, at age 9–11, was obtained from the National Population and Housing Census of 1960. Socioeconomic position was based on the occupation of the head of the household, most often the father. Occupation was classified into six socioeconomic groups: 1. unskilled workers; 2. skilled workers; 3. assistant non-manual employees; 4. non-manual employees at intermediate or higher level; 5. farmers; 6. those not classified in a socioeconomic group. Crowded housing was classified as >2 people/room (kitchen not included).

#### Factors measured at conscription

All men underwent a medical examination and a psychological interview. Height was recorded and is used in the analysis as a proxy for circumstances in childhood, as done previously [33]. Height was dichotomized (above or below 170 cm) when showing its associations with suicide/attempted suicide (tables 1, 2 and S1); otherwise, height was included as a continuous variable (tables 3–5 and S2).

**Table 1:** Prevalence of potential explanatory factors stratified by WHO categories of BMI.

	N exposed	<18.5 (underweight, n = 6739)	18.5–25 (normal weight, n = 38930)	25–30 (overweight, n = 2832)	>30 (obese, n = 403)
**Early life factors**					
Low childhood SEP	26 898	52.60	54.15	62.04	66.00
Crowded housing	10 054	21.27	20.43	22.03	25.94
Short stature	5 382	10.37	10.96	12.39	16.63
**Mental health and life style factors at conscription**					
Low emotional control	14 927	35.92	29.35	30.23	32.59
Psychiatric diagnosis, conscription	5 691	14.56	10.70	10.98	12.66
Depression diagnosis, conscription	635	1.57	1.22	1.27	0.74
Depressed mood a	10 451	23.82	21.06	19.01	17.09
Smoking	28 424	63.30	57.73	57.36	61.01
Risky use of alcohol	6 422	11.28	13.71	15.24	12.30
**Mental health and socioec./social factors in early adulthood**					
Psychiatric diagnosis, hospital discharge 1973–80	1 638	3.72	3.19	2.79	2.23
Depression diagnosis, hospital discharge 1973–80	318	0.77	0.64	0.39	0.00
Manual Worker 1980	22 327	43.39	44.84	53.50	60.55
Unmarried/living alone 1980	15 549	37.87	30.60	34.20	50.76

Abbreviations: BMI, Body Mass Index; N, Number; SEP, Socio-Economic Position; WHO, World Health Organization.

a Feeling “down” more than occasionally.

**Table 2:** Associations between potential explanatory factors and attempted and completed suicide 1973/1971–2008.

	Attempted suicide		Completed suicide	
	**HR**	95% CI	**HR**	95% CI
Low childhood SEP (manual occupation of head of household)	**1.41**	1.25–1.58	**0.98**	0.84–1.15
Crowded housing	**1.70**	1.50–1.92	**1.50**	1.26–1.78
Short stature (<170 cm)	**1.35**	1.15–1.58	**1.65**	1.34–2.03
Low emotional control (1–2)	**2.70**	2.41–3.02	**2.04**	1.75–2.38
Psychiatric diagnosis, conscription	**3.45**	3.05–3.90	**2.62**	2.19–3.14
Depressed mood	**1.95**	1.73–2.20	**1.87**	1.58–2.20
Smoking	**2.67**	2.33–3.07	**1.76**	1.48–2.09
Risky use of alcohol	**3.50**	3.11–3.95	**2.62**	2.20–3.13
Psychiatric diagnosis, hospital discharge, 1973–80^a^	**14.56**	12.51–16.96	**8.95**	7.03–11.41
Depression diagnosis, hospital discharge, 1973–80^a^	**11.16**	8.10–15.39	**5.23**	2.79–9.79
Manual occupation 1980^a^ ^b^	**2.02**	1.72–2.37	**1.65**	1.33–2.05
Unmarried/living alone 1980^a^	**1.84**	1.61–2.10	**2.09**	1.73–2.52

Abbreviations: CI, Confidence Interval; HR, Hazard Ratio; SEP, Socio-economic position.

a Follow-up from 1981.

b Excluding men with no recorded occupation.

**Table 3:** Associations between BMI at conscription and attempted suicide 1973–2008 and completed suicide 1971–2008; crude and adjusted hazard ratios per one standard deviation increase in BMI.

	Attempted suicide		Completed suicide	
Total n (cases)	45 365 (1136)		45 454 (590)	
	**HR**	95% CI	**HR**	95% CI
Crude	**0.88**	0.83–0.94	**0.93**	0.85–1.01
Adjusted for:				
Childhood SEP	**0.88**	0.82–0.93	**0.93**	0.85–1.01
Crowded housing	**0.88**	0.83–0.93	**0.93**	0.85–1.01
Height	**0.87**	0.82–0.93	**0.92**	0.84–1.00
Emotional control	**0.91**	0.86–0.97	**0.95**	0.88–1.03
Psychiatric diagnosis, conscription	**0.90**	0.85–0.96	**0.94**	0.87–1.03
Depressed mood	**0.89**	0.84–0.95	**0.94**	0.86–1.02
Risky use of alcohol	**0.86**	0.80–0.91	**0.92**	0.84–0.99
Smoking	**0.90**	0.84–0.95	**0.94**	0.86–1.02
All variables	**0.89**	0.84–0.95	**0.94**	0.86–1.02

Abbreviations: BMI, Body Mass Index; CI, Confidence Interval; HR, Hazard Ratio; N, Number; SEP, Socio-Economic Position.

**Table 4:** Associations between BMI at conscription and attempted and completed suicide 1981–2008^a^; crude and adjusted hazard ratios per one standard deviation increase in BMI.

	Attempted suicide		Completed suicide	
Total n (cases)	44 440 (821)		44 440 (408)	
	**HR**	95% CI	**HR**	95% CI
Crude	**0.89**	0.83–0.96	**0.94**	0.85–1.04
Adjusted for:				
All earlier variables^b^	**0.91**	0.84–0.97	**0.94**	0.85–1.04
Psychiatric diagnosis 1973–80	**0.92**	0.85–0.98	**0.96**	0.87–1.06
SEP 1980	**0.89**	0.83–0.96	**0.94**	0.85–1.04
Unmarried/living alone 1980	**0.90**	0.84–0.96	**0.95**	0.86–1.04
All variables in adulthood	**0.91**	0.85–0.98	**0.96**	0.87–1.06
All variables^c^	**0.93**	0.86–1.00	**0.96**	0.87–1.06

Abbreviations: BMI, Body Mass Index; CI, Confidence Interval; HR, Hazard Ratio; N, Number; SEP, Socio-Economic Position.

a Men who died before 1981 or have missing data on any variable recorded in adulthood are excluded; thus, estimates are slightly different from the analyses presented in table 3.

b Adjusted for childhood SEP, crowded housing, height, emotional control, risky use of alcohol, smoking, depressed mood, and psychiatric diagnosis at conscription.

c Adjusted for all factors in childhood, at conscription and in adulthood.

**Table 5:** Associations between BMI at conscription, in WHO categories of BMI, and attempted suicide 1973/1981–2008 and completed suicide 1971/1981–2008.

	<18.5		18.5–25	25–30		>30	
	HR	95% CI	Reference	HR	95% CI	HR	95% CI
Attempted suicide 73–08	*6210 (196)* a		*36166 (878)* a	*2630 (56)* a		*359 (6)* a	
Crude	**1.30**	1.12–1.52	1	**0.88**	0.67–1.15	**0.70**	0.32–1.57
Adjusted^b^	**1.20**	1.03–1.40	1	**0.84**	0.64–1.11	**0.66**	0.29–1.46
Attempted suicide 81–08	*6072 (141)* a		*35398 (634)* a	*2578 (41)* a		*352 (5)* a	
Crude	**1.30**	1.09–1.56	1	**0.88**	0.64–1.21	**0.80**	0.33–1.93
Adjusted^b^	**1.14**	0.95–1.37	1	**0.82**	0.60–1.13	**0.64**	0.27–1.56
Completed suicide 71–08	*6222 (81)* a		*36239 (479)* a	*2632 (25)* a		*361 (5)* a	
Crude	**0.98**	0.78–1.24	1	**0.72**	0.48–1.08	**1.08**	0.45–2.60
Adjusted^c^	**0.94**	0.74–1.19	1	**0.71**	0.48–1.07	**1.06**	0.44–2.57
Completed suicide 81–08	*6072(61)* a		*35398 (323)* a	*2578 (20)* a		*352(4)* a	
Crude	**1.10**	0.84–1.45	1	**0.86**	0.55–1.34	**1.28**	0.48–3.42
Adjusted^c^	**1.01**	0.77–1.33	1	**0.83**	0.53–1.31	**1.16**	0.43–3.11

Abbreviations: BMI, Body Mass Index; CI, Confidence Interval; HR, Hazard Ratio; SEP, Socio-Economic Position; WHO, World Health Organization.

a Number in category and (cases).

b Adjusted for childhood SEP, crowded housing, height, emotional control, risky use of alcohol, smoking, depressed mood, and psychiatric diagnosis at conscription.

c Adjusted for childhood SEP, crowded housing, height, emotional control, risky use of alcohol, smoking, depressed mood, psychiatric diagnosis at conscription and from hospital admission 1973–80, and SEP and marital status in 1980.

In any case of reported or suspected psychiatric illness, the conscript was examined by a psychiatrist who recorded any mental disorder in accordance with the ICD-8, the diagnostic system used in Sweden at that time. Emotional control was assessed in a structured interview by a trained psychologist, and rated on a scale from one to five based on the conscripts' responses to questions about behaviors and strategies in various types of situations. A low rating was given to conscripts who seemed to lack an ability to control nervousness and anxiousness, channel aggression, commit emotionally, and/or had documented functional limitations due to psychosomatic symptoms. A high rating was given to conscripts who seemed able to act calmly in most kinds of situations. This variable has previously been shown to be associated with suicide in this cohort [27], [34]. The psychologists' ratings were regularly tested for inter-rater reliability [35].

The conscripts provided information on social background, behavior and adjustment, health, substance use, and psychological and psychosomatic symptoms in questionnaires. A question of how often they felt “down” (often, sometimes, occasionally or never) was included. This variable has previously been used as a subclinical measure of depressed mood [36], and has been shown to be associated with suicidal behavior [33]. Smoking was classified in categories of no smoking, smoking 1–10, 11–20 and >20 cigarettes per day. A composite variable, ‘risky use of alcohol’, was created from affirmative answers to one or more of the following: consumption of >250 g 100% alcohol/week, reporting drinking alcohol as an ‘eye-opener’ during a hangover, having been apprehended for drunkenness, or having ‘often’ been drunk (other choices were ‘rather often’, ‘sometimes’, and ‘never’). Frequency of drug use (illicit/illegal drugs) was classified as never, once, 2–4, 5–10, 11–50, or more than 50 times.

#### Factors in adulthood

Information on psychiatric diagnoses (ICD 8 codes 395–315), recorded after hospital admission for any conditions between 1973 and 1980, was obtained from the National Hospital Discharge Register. Information on socioeconomic position and marital status at 34–36 years of age was obtained from the National Population and Housing Census of 1980. The classification into eight socioeconomic groups was based on occupation: 1. unskilled workers; 2. skilled workers; 3. assistant non-manual employees; 4. non-manual employees at intermediate level; 5. non-manual employees at higher level; 6. farmers; 7. self-employed (mostly skilled workers or drivers); 8. those for whom no occupation was reported (e. g. unemployed, early retired or disabled). Marital status was dichotomized as married/cohabiting or unmarried/living alone (including divorced and widowed men).

### Statistical analysis

The associations of BMI at conscription with suicide 1971–2008 and suicide attempt 1973–2008 were estimated using Cox's proportional hazard models in SAS 9.2, adjusting for potential explanatory variables from childhood and adolescence separately and in combination. Proportional hazards were assessed with log (−log) survival plots. BMI was modelled as a continuous varible with hazard ratios (HR) given per standard deviation (SD) increase. Tests of linear associations were conducted by comparing models with and without the quadratic term, and the model without the quadratic term was found to fit the data best.

A HR higher than one indicates a higher risk with increasing BMI, while a HR lower than one indicates a lower risk. If the 95% confidence interval (CI) does not include one, the association is statistically significant at the 0.05-level. HRs were also calculated after categorizing BMI according to the WHO classifications. The group of men with normal weigth was treated as the reference group. HRs for 5th, 10th, 25th, 75, 90th and 95th percentiles of BMI, with the 25th-75th percentile as the reference group, were also calculated (table S2). Dependent variables were suicide mortality and first suicide attempt. Censoring was made at the time of the event, emigration, or December 31th 2008, whichever occurred first. The analyses were repeated with follow-up from 1981, adding adulthood adjustment variables recorded up until 1980. Sensitivity analyses were performed excluding cases with undetermined intent (retaining only ICD codes E950-9 [ICD-8/9] and X60–X84 [ICD-10]).

## Results

Of the 49 321 men born 1949–1951 who were conscripted in 1969/70, there were 45 454 (92.2%) men who had information on all variables. These men were included in the main analyses for the full follow up-period with completed suicide as outcome (1971–2008). Attempted suicide was recorded from 1973, and the 89 men who died before that left 45 365 (92.0%) men for analysis. The follow-up from 1981 included 44 440 (90.1%) men.

Table 1 shows the prevalence of potential explanatory factors across the WHO's BMI categories. The prevalence for the following variables was highest among men who were underweight at conscription: depression or other psychiatric diagnosis at conscription or in early adulthood; low emotional control; depressed mood (feeling “down” more than occasionally); and smoking. There was some evidence of a reversed J-shaped association (high incidence in obese men) for all psychiatric diagnosis at conscription, low emotional control, and smoking. Low socioeconomic position in childhood or adulthood became more common with increasing BMI group, with the highest prevalence among men who were obese at conscription. The prevalences of potential explanatory factors in groups based on percentiles of BMI are shown in table S1.

Table 2 shows the associations of the potential explanatory factors with attempted and completed suicide. Hospital admission with a psychiatric diagnosis in early adulthood was associated with the highest risk of later incidents (HR for suicide: 8.95 and for suicide attempts: 14.56). Recieving a psychiatric diagnosis and reporting risky use of alcohol at conscription were also among the strongest risk factors.

Table 3 shows the associations of risk factors with attempted and completed suicide during 36 and 38 years of follow-up, respectively. During follow-up, there were 1136 cases of attempted suicide and 590 cases of completed suicide in the analytical samples. One standard deviation (SD, corresponding to 2.560 kg/m^2^) increase in BMI was associated with a 12% lower risk of attempted suicide, while the association was weaker and did not reach conventional levels of statistical significance for completed suicide. Emotional control, assessed at conscription, had modest attenuating impact on the association of BMI with attempted suicide, while adjusting for risky use of alcohol had a strengthening impact. Adjusting for all factors in the full model had little effect on the estimate.

Table 4 shows the associations of BMI with attempted and completed suicide between 1981 and 2008, controlling for factors in adulthood. These factors had only marginal impact on the estimates. Additional adjustment for all earlier factors did not change the estimates.

Table 5 shows the association with attempted and completed suicide for WHO categories of BMI, compared to normal weight as reference. Being underweight at conscription was associated with a 30% increased risk of attempted suicide during the full follow-up period. The association was attenuated in the full model, but a statistically significant increased risk of 20% remained. The HRs decreased incrementally with increasing BMI. Regarding completed suicide, the pattern was inconsistent and confidence intervals were wide. Adjustment for somatic diagnoses at conscription had no effect on the estimates.

HRs for the lowest (5th, 10th, 25th) and highest (75th, 90th and 95th) percentiles of BMI, with the 25th–75th percentile as the reference group, showed a similar pattern. Men in the lower BMI percentiles had an increased risk of attempted and completed suicide, although few estimates reached the conventional level of statistical significance (table S2). Adjusting for history of drug use at the time of conscription (number of times) in a subsample of 43 297 men with such data attenuated the estimates minimally (data available on request).

Excluding deaths of undetermined intent yielded similar estimates for HRs per SD increase in BMI in crude and fully adjusted models. However, regarding attempted suicide, analyses on groups according to the WHO classifications revealed a stronger association among men who were underweight at conscription and a somewhat weaker association among men with overweight, compared to analyses including undetermined cases. Regarding completed suicide, the trend was rather a lowering of the estimate among both underweight and overweight men, although none of these estimates were statistically significant.

## Discussion

### Main findings

In this large cohort of Swedish male conscripts, body mass index (BMI) in early adulthood was inversely associated with later risk of hospital admission following a suicide attempt; there was little evidence that this association was confounded by a range of social, behavioral and psychological risk factors for suicide. There was only limited evidence of an inverse association with suicide, although the direction of association is consistent with that seen in previous studies and the 95% confidence intervals overlap with those previously documented [1], [2], [3], [4], [5], [6]. In contrast to many previous studies [6], [19], our indicators of depression (hospital admission/clinical diagnosis at baseline) were highest in those who were underweight in early adulthood, although there was also some evidence of increased risk amongst the obese.

### Attempted suicide

Our findings are similar to those reported in the few previous longitudinal studies to date [2], [9], [30]. In a larger cohort of Swedish conscripts (overlapping with the cohort used in this study but with fewer variables available), the association of BMI with attempted suicide was slightly weaker and only present after adjustment for socioeconomic position and education (HR 0.93, 95% CI 0.91–0.94 per SD increase) [9]. Unadjusted models showed a U-shaped association with higher HRs in the underweight and obese groups, and no difference in the overweight group, compared to men with normal weight (only the adjusted model was linear). This study did not control for psychiatric or somatic diagnoses, emotional control, mood or substance use [9]. In the Danish Metropolit cohort, comprising men born 1953, BMI measured at age 18 was inversely associated with attempted suicide up to age 49 with the HR 0.86 (95% CI 0.74–0.99) per SD increase [2]. The estimate changed minimally after adjustment for e. g. childhood socioeconomic position, cognitive ability at age 12, education and mental disorder at conscription. These results are similar to our findings. Among British male patients in primary care, Gao et al [30] found a similar trend in analyses of WHO-classified BMI groups.

### Completed suicide

In contrast to most previous studies, we did not find strong evidence for an association between BMI at conscription and completed suicide. In a larger cohort of Swedish conscripts, the association of BMI with completed suicide was similar to that seen in our analyses. Each 5-unit increase in BMI (corresponding to approximately 2 SD) was associated with a HR of 0.85, 95% CI 0.79–0.91 [1]. In that study, adjustments for psychiatric diagnosis at conscription, and parental socioeconomic position and education, had little effect on the estimate. No adjustments were made for emotional control, mood, substance use or somatic disorders at conscription.

Most other longitudinal studies have found associations of even greater magnitudes [2], [3], [4], [5], [6], [37]. For example, the Danish Metropolit cohort study described above reported a HR of 0.79 (95% CI 0.61–1.00) per SD increase of BMI [2]. In line with our findings, some studies have failed to find a statistically significant association between lower BMI and later suicide [7], [30]. Chang et al reported a statistically significant U-shaped association, but not a linear one [29]. Overall, associations have been robust in multivariable models including e. g. smoking, alcohol use, comorbidity and disease history [4], [5], [29], socioeconomic background [1], [2], education [2], [6], [29], marital status [4], [6], [29], psychiatric symptoms and conditions [1], [2], [6], physical activity [4], [5], [6], [37], and biological markers such as blood glucose and blood cholesterol [37]. One exception is a study on a large Chinese sample, in which adjustments including employment status, education and living in a rural area explained the larger part of the inverse association between BMI and suicide [38].

Our findings differ from the pattern reported by Zhang et al [20] of a stronger and more consistent inverse association with more severe suicidal behaviors. There are several possible explanations for our divergent findings. First, it might be a chance finding related to lack of statistical power; the 95% CIs overlap with previous findings. Second, there might be a cohort effect. It is possible that differences in culture and lifestyle between cohorts contribute to the different results, e. g. changes in eating habits and in body image ideals. Third, the age at BMI measurement might be important. The conscripts in our study are aged 18–20, while most other study populations are older or have larger age spans. It is possible that BMI in youth is differently related to mechanisms related to suicidal behavior, e. g. regarding mental health.

### Psychological factors

In the Nord-Trøndelag Health (HUNT) study, although higher BMI was associated with lower suicide risk, higher BMI also predicted depression on the following 21 years [6]. In contrast, we found that the incidence of psychiatric illness leading to hospital admission declined with increasing BMI. Psychiatric diagnoses, psychologists' rating of low emotional control, and self-reported depressed mood at conscription were also more common among underweight men. Thus, the paradox reported in the HUNT study was not replicated in this cohort. Differences in the characteristics of hospital-presenting and community identified depression may account for differences in the associations. In the HUNT cohort, depression was measured using the Hospital Anxiety and Depression Scale (HADS) – a screen for depression – and the prevalence of apparent disorder was high (>10%) whereas in our study it was 1.2% at conscription and the incidence in hospital records 1973–1980 was <1%. However, self-reported depressed mood at the time of conscription also had an inverse association with BMI in our study, suggesting that severity does not entirely explain this difference. Furthermore, differences between the cohorts regarding e. g. demographics and time period might contribute to the diverse results. It is possible that mechanisms linking BMI to psychological distress differ between young men in the 1970's and adult men and women (mean age 48) almost two decades later. For instance, in a meta-analysis the associations between overweight and later depression were found only among adults aged 20 or more, and not in younger populations [10].

### Possible mechanisms

We investigated some suggested explanations [19], [20] for the association between BMI and suicidal behavior: possible confounding by pre-existing psychiatric or somatic illness, socioeconomic position, smoking and substance misuse; and later psychiatric illness as indicated by hospitalization with a psychiatric diagnosis. We were also able to control for emotional control, a personality trait which has not been included in previous studies. There was little evidence that controlling for any of these factors substantially altered the strength of the associations we observed. Controlling for risky use of alcohol strengthened the association, indicating that failure to control for alcohol misuse may lead to an under-estimation of the association between BMI and suicidal behavior.

Other explanations for the association have been suggested. Genetic covariance might act as a confounder; biological factors associated with body weight and adiposity tissue, such as insulin resistance, leptin levels and neuroendocrine processes, might in various ways affect the risk of suicidal behavior; a higher intake of dietary carbohydrates might prevent depressive symptoms and suicides, and also lead to weight gain; an increased body size might lead to lower case-fatality of attempted suicides (or lower rates of attempts leading to overnight hospital care, in our study); underweight might be associated with a negative body image among men; weight-affecting medication and other substance use, including smoking, might confound the association; and pre-existing somatic diseases might also act as confounders [19], [20]. Since adjusting for somatic diagnoses at conscription had no effect on the association between BMI and suicide attempt, the latter does not appear to explain the association in this study. Neither did confounding by substance misuse or smoking. We had no data on neurobiological or genetic factors, diet, body image, or rate of attempts leading to hospital care in this cohort.

### Strengths and limitations

We obtained objective measures of BMI from a medical screening examination carried out on >95% of Swedish men aged ∼18 years. Our measures are therefore not likely to be influenced by reporting biases or the impact on body weight of comorbid chronic diseases (e.g. diabetes, osteoarthritis) whose prevalence increases with age. Furthermore, we were able to control for a unique set of variables including psychiatric diagnoses from the medical examination at conscription as well as from hospital records in early adulthood.

The homogeneity by age, sex and ethnicity of the cohort may limit its generalizability to other populations and females. Moreover, participants were measured in 1969–70, over a decade before the epidemic increases in obesity seen in many industrialised countries. For instance, between 1971 and 1995 the prevalence of overweight at conscription doubled, and for obesity almost quadrupled [39]. The small number of cases among men who were obese at conscription causes low power and uncertain results in this group. Men who were very obese (BMI>35), and might have an increased risk of suicidal behavior [29], were too few to analyse separately which might have diluted the estimate in the obese group. Categories by WHO definitions yield very unevenly numbered groups in this data. The majority were classified as normal weight and the cases of attempted and completed suicide are very low in some of the other categories.

Self-reports of measures such as drinking habits and mood are sensitive to possible reporting bias, and this may influence some of the associations we observed if any under/over-reporting is differential in relation to an individual's weight. Similarly, the psychologists' assessments of emotional functioning might be influenced by a conscript's weight and physical appearance. However, a detailed manual was used for the assessment and the inter-rater reliability was high (r = 0.86; measured at conscription in 1972–73[35]). Furthermore, all measures of the conscripts' psychological state, i. e. self-reports, psychologist's assessment, psychiatric diagnoses from conscription and diagnoses from hospital records, point in the same direction in relation to BMI in this study.

### Conclusion

We found a graded association of higher BMI in young adulthood with lower risk of attempted suicide up to age 59. The association was little affected by adjustment for socioeconomic factors, psychiatric diagnoses and substance use. No association of BMI with completed suicide was evident. The previously reported paradox of higher BMI being associated with lower risk of suicidal behavior but higher risk of mental ill-health was not found in this study, since having a psychiatric diagnosis and low emotional control were also associated with lower BMI. There is a need to further understand the biological basis of the association of BMI with suicidal behavior as this may provide important pointers to prevention and treatment.

## Supporting Information

Table S1 Prevalence of potential explanatory factors by percentiles of BMI.
(DOCX)

Table S2 Associations between BMI at conscription, by percentiles of BMI, and attempted suicide 1973/1981–2008 and completed suicide 1971/1981–2008.
(DOCX)
