# Supplementary material for: Body Mass Index in Young Adulthood and Suicidal Behavior up to Age 59 in a Cohort of Swedish Men
Source: PLoS One. 2014 Jul 1;9(7):e101213. doi: 10.1371/journal.pone.0101213 (PMC4077734; doi:10.1371/journal.pone.0101213)
Supplement: Table S1 — Prevalence of potential explanatory factors by percentiles of BMI. (DOCX) [file pone.0101213.s001.docx]

Table S1. Prevalence of potential explanatory factors by percentiles of BMI.

|  |  | **BMI percentiles**  mean*, range* | | | | | | |
| --- | --- | --- | --- | --- | --- | --- | --- | --- |
|  |  | **≤5th p.**  16.8,  *12.9-17.5* | **≤10th p.**  17.3,  *12.9-18.1* | **≤25th p.**  18.2,  *12.9-19.3* | **25-75th p.**  20.7,  *19.3-22.2* | **≥75th p.**  24.4,  *22.2-44.6* | **≥90th p.**  26.3,  *24.1-44.6* | **≥95th p.**  27.9,  *25.6-44.6* |
|  | N exposed | % | % | % | % | % | % | % |
| **Early life factors** |  |  |  |  |  |  |  |  |
| Low childhood SEP | 26 635 | 53.72 | 52.04 | 52.76 | 53.50 | 58.18 | 61.21 | 63.27 |
| Crowded housing | 9 946 | 21.56 | 21.32 | 21.04 | 20.16 | 21.37 | 22.49 | 23.09 |
| Short stature | 5 365 | 10.87 | 9.93 | 10.59 | 10.66 | 11.99 | 12.49 | 13.06 |
| **Mental health and life style factors at conscription** |  |  |  |  |  |  |  |  |
| Low emotional control | 14 755 | 40.57 | 37.12 | 34.64 | 29.05 | 28.57 | 29.55 | 31.42 |
| Psychiatric diagnosis, conscription | 5 505 | 17.21 | 15.21 | 13.47 | 10.65 | 10.27 | 11.04 | 11.92 |
| Depression diagnosis, conscription | 620 | 1.92 | 1.65 | 1.47 | 1.21 | 1.19 | 1.32 | 1.19 |
| Depressed mood ^a^ | 10 292 | 25.89 | 24.38 | 23.03 | 21.24 | 19.65 | 19.31 | 18.58 |
| Smoking | 28 175 | 63.64 | 63.45 | 62.68 | 57.99 | 55.37 | 56.61 | 58.47 |
| Risky use of alcohol | 6 310 | 9.87 | 11.00 | 11.67 | 13.36 | 15.41 | 15.04 | 15.17 |
| **Mental health and socioec./ social factors in early adulthood** |  |  |  |  |  |  |  |  |
| Psychiatric diagnosis, hospital discharge 1973-80 | 1 581 | 4.01 | 3.93 | 3.76 | 3.18 | 2.82 | 2.75 | 3.03 |
| Depression diagnosis, hospital discharge 1973-80 | 311 | 0.74 | 0.82 | 0.74 | 0.68 | 0.45 | 0.37 | 0.33 |
| Manual Worker 1980 | 22 126 | 43.58 | 42.70 | 44.02 | 44.14 | 48.78 | 52.91 | 55.77 |
| Unmarried/living alone 1980 | 15 348 | 41.90 | 38.98 | 35.73 | 30.48 | 31.19 | 34.67 | 38.19 |

Abbreviations: BMI, Body Mass Index; N, Number; SEP, Socio-Economic Position.

^a^ Feeling ”down” more than occasionally.
