# Supplementary material for: Body Mass Index in Young Adulthood and Suicidal Behavior up to Age 59 in a Cohort of Swedish Men
Source: PLoS One. 2014 Jul 1;9(7):e101213. doi: 10.1371/journal.pone.0101213 (PMC4077734; doi:10.1371/journal.pone.0101213)
Supplement: Table S2 — Associations between BMI at conscription, by percentiles of BMI, and attempted suicide 1973/1981–2008 and completed suicide 1971/1981–2008. (DOCX) [file pone.0101213.s002.docx]

Table S2. Associations between BMI at conscription, by percentiles of BMI, and attempted suicide 1973/1981-2008 and completed suicide 1971/1981-2008.

|  | **≤5th p.** | | **≤10th p.** | | **≤25th p.** | | **25-75th p.** | **≥75th p.** | | **≥90th p.** | | **≥95th p.** | | |
| --- | --- | --- | --- | --- | --- | --- | --- | --- | --- | --- | --- | --- | --- | --- |
|  | **HR** | **95% CI** | **HR** | **95% CI** | **HR** | **95% CI** | **Reference** | **HR** | **95% CI** | **HR** | **95% CI** | **HR** | **95% CI** | |
| Attempted suicide 73-08 | *2238 (74)^a^* | | *4519 (148) ^a^* | | *11259 (336) ^a^* | | *11259 (553) ^a^* | *11372 (247) ^a^* | | *4569 (103) ^a^* | | *2266 (47) ^a^* | | |
| Crude | **1.37** | 1.07-1.74 | **1.36** | 1.13-1.63 | **1.23** | 1.08-1.41 | **1** | **0.90** | 0.77-1.04 | **0.94** | 0.76-1.16 | **0.87** | | 0.64-1.17 |
| Adjusted^b^ | **1.24** | 0.97-1.59 | **1.25** | 1.04-1.51 | **1.16** | 1.01-1.32 | **1** | **0.88** | 0.76-1.02 | **0.90** | 0.73-1.11 | **0.81** | | 0.60-1.09 |
| Attempted suicide 81-08 | *2194 (54) ^a^* | | *4416 (106) ^a^* | | *11008 (240) ^a^* | | *22262 (398) ^a^* | *11130 (183) ^a^* | | *2281 (80) ^a^* | | *2215 (34) ^a^* | | |
| Crude | **1.39** | 1.04-1.84 | **1.35** | 1.09-1.67 | **1.22** | 1.04-1.44 | **1** | **0.92** | 0.77-1.10 | **1.01** | 0.80-1.29 | **0.87** | | 0.62-1.24 |
| Adjusted^b^ | **1.16** | 0.87-1.54 | **1.17** | 0.94-1.45 | **1.11** | 0.95-1.31 | **1** | **0.94** | 0.79-1.13 | **1.00** | 0.79-1.28 | **0.80** | | 0.56-1.14 |
| Completed suicide 71-08 | *2246 (37) ^a^* | | *4530 (67) ^a^* | | *11278 (156) ^a^* | | *22779 (289) ^a^* | *11397 (145) ^a^* | | *4579 (47) ^a^* | | *2268 (27) ^a^* | | |
| Crude | **1.30** | 0.93-1.84 | **1.16** | 0.89-1.52 | **1.09** | 0.90-1.33 | **1** | **1.01** | 0.83-1.23 | **0.77** | 0.56-1.06 | **0.96** | | 0.65-1.42 |
| Adjusted^c^ | **1.28** | 0.91-1.81 | **1.15** | 0.88-1.50 | **1.06** | 0.87-1.28 | **1** | **1.00** | 0.82-1.23 | **0.77** | 0.56-1.05 | **0.95** | | 0.64-1.41 |
| Completed suicide 81-08 | *2194 (27) ^a^* | | *4416 (50) ^a^* | | *11008 (108) ^a^* | | *22262 (197) ^a^* | *11130 (103) ^a^* | | *4481 (38) ^a^* | | *2215 (21) ^a^* | | |
| Crude | **1.39** | 0.93-2.08 | **1.27** | 0.93-1.73 | **1.11** | 0.88-1.40 | **1** | **1.05** | 0.83-1.33 | **0.90** | 0.63-1.29 | **1.09** | | 0.70-1.71 |
| Adjusted^c^ | **1.24** | 0.82-1.86 | **1.18** | 0.86-1.61 | **1.04** | 0.82-1.32 | **1** | **1.06** | 0.84-1.35 | **0.88** | 0.61-1.26 | **1.02** | | 0.65-1.61 |

Abbreviations: BMI, Body Mass Index; CI, Confidence Interval; HR, Hazard Ratio; SEP, Socio-Economic Position.

^a^ Number in category and (cases).

^b^ Adjusted for childhood SEP, crowded housing, height, emotional control, risky use of alcohol, smoking, depressed mood, and psychiatric diagnosis at conscription.

^c^ Adjusted for childhood SEP, crowded housing, height, emotional control, risky use of alcohol, smoking, depressed mood, psychiatric diagnosis at conscription and from hospital admission 1973-80, and SEP and marital status in 1980.
